# A Review of the Associations Between Obstructive Sleep Apnea and Gestational Diabetes Mellitus and Possible Mechanisms of Disease

**DOI:** 10.1007/s43032-022-00904-3

**Published:** 2022-03-07

**Authors:** Xingyi Tong, Linzhu Yang, Chengyan Jiang, Zhiying Weng, Anju Zu, Yunjiao Hou, Yan Fang, Weimin Yang, Shibo Sun

**Affiliations:** 1 Department of Pulmonary and Critical Care Medicine, First Affiliated Hospital, Kunming Medical University, Kunming, China; 2 Clinical Medicine & Pediatrics, 2017 Grade, Kunming Medical University, Kunming, China; 3 Thoracic Surgery, First Affiliated Hospital, Kunming Medical University, Kunming, China; 4 School of Pharmaceutical Science & Yunnan Key Laboratory of Pharmacology for Natural Products, Kunming Medical University, Kunming, China

**Keywords:** Obstructive sleep apnea, Sleep disorders, Gestational diabetes mellitus, Pregnancy, Obesity

## Abstract

Obstructive sleep apnea (OSA) usually leads to the occurrence of diabetes. Gestational diabetes mellitus (GDM) is a common gestational complication associated with adverse maternal and fetal outcomes. Increasing studies suggest that women with OSA during pregnancy may be at a significantly greater risk of developing GDM. It is crucial to explore the association between OSA and GDM and the mechanisms underlying this association. In this review, we presented a comprehensive literature review of the following: the association between OSA and GDM, the possible mechanisms of this association, and the effects of continuous positive airway pressure (CPAP) on OSA with GDM. The results showed that most authors suggested that there was an association between OSA and GDM. The intermittent hypoxemia (IH) and reduction of slow-wave sleep (SWS) may be the key to this association. IH induces the products of oxidative stress and inflammation as well as dysregulation of the hypothalamic–pituitary–adrenal, which lead to diabetes. In addition, SWS reduction in OSA enhances the inflammation by increasing the inflammatory cytokines, increases the sympathetic activation, and causes changes in leptin level, which result in the development of GDM. Additionally, whether CPAP is beneficial to GDM remains still unclear.

## Introduction

Obstructive sleep apnea (OSA) is characterized by recurrent intermittent hypoxemia (IH) and arousals during sleep. It is reported that OSA impairs glucose-induced insulin secretion from pancreatic β cells and causes the aggregation of inflammatory factors in adipose tissue, leading to the occurrence of diabetes or insulin resistance (IR) [1, 2]. Continuous positive airway pressure (CPAP), as the gold standard for OSA treatment, improves diabetes or IR in OSA patients [3, 4]. Consequently, there is an association between OSA and diabetes, and this association is independent of adiposity and family history of diabetes [5, 6].

As a state of abnormal glucose tolerance, gestational diabetes mellitus (GDM) is a common gestational complication defined as any degree of glucose intolerance with onset or first recognition during pregnancy [7]. GDM is usually diagnosed at 24–28 weeks of pregnancy and is associated with adverse maternal and fetal outcomes [8–12]. In addition, mothers with GDM have an increased risk of preeclampsia, cesarean section, premature delivery, polyhydramnios, and infection [13–15]. The infants of a diabetic mother have an increased risk of neurodevelopmental deficits or physical defects [13, 16].

The associations between OSA and GDM may not be causal. Increasing evidence suggest that OSA is associated with GDM [17, 18]. It is reported that the prevalence of OSA is 3.6–22% in different stages of pregnancy [17, 19, 20]. Bisson M. et al. estimated the prevalence of OSA in GDM is about 31% [21]. In addition, Facco FL. et al. found “high-risk” women (BMI ≥ 30 kg/m^2^, hypertension, pre-pregnancy diabetes, preeclampsia, and/or twin pregnancy) had a much higher incidence of OSA from early pregnancy to the third trimester of pregnancy, ranging from 30 to 50% [22].

The mechanisms that link OSA to GDM are not yet clear. Moreover, continuous positive airway pressure (CPAP) is the gold standard treatment for OSA. So far, few studies have systematically explored the effect of CPAP on GDM with OSA. This article aimed to review the association between OSA and GDM, the possible mechanisms of association between OSA and GDM, and the effect of CPAP on OSA with GDM.

## Methods

**Fig. 1:**
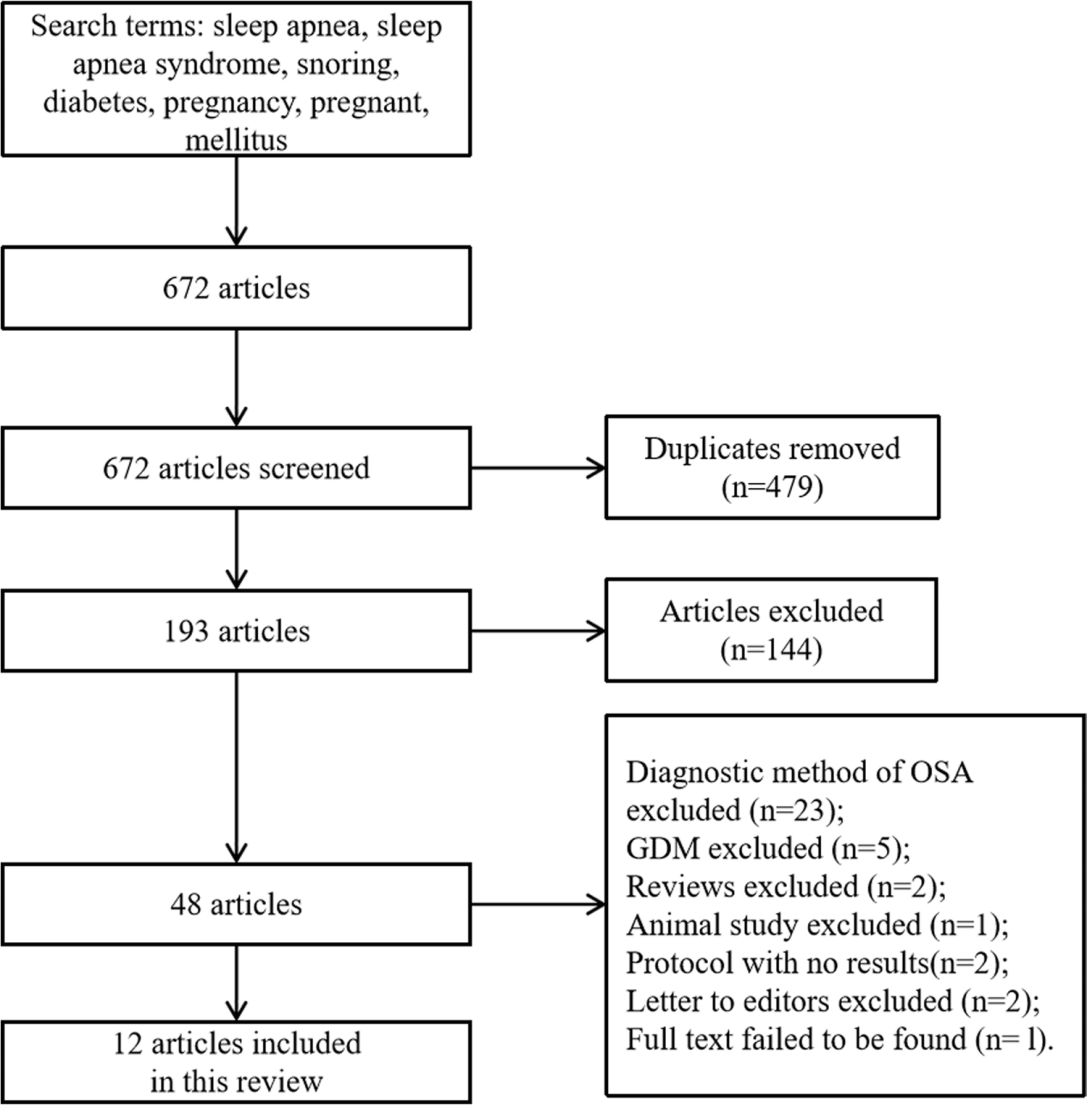
Chart of article screened strategy. *OSA*, obstructive sleep apnea; *GDM*, gestational diabetes mellitus

PubMed was used to search the articles published between March 1, 1977, and August 24, 2021. Search terms in PubMed included sleep apnea, sleep apnea syndrome, snoring, diabetes, pregnancy, pregnant, and mellitus. The articles were included, in which the OSA was diagnosed with polysomnography (PSG), home sleep testing monitor, or Watch-PAT, rather than symptom-based questionnaires, as some studies suggested that these questionnaires were not credible among pregnant women [23–25]. In addition, literature in languages other than English were excluded as well as the conference abstract or literature. The chart of the article screened is shown in Fig. 1.

## Results

Twelve articles were finally included in this review (Tables 1 and 2), of which nine were prospective and three were case–control studies. In addition, seven articles included were published in the last five years. Among these articles, there were three randomized controlled trials (RCT) [26–28].

**Table 1:** Selective articles on OSA with GDM

Reference	Type of study	Number of subjects	Median BMI or mean ± SD	Diagnostic criteria for OSA (events/h)	Gestational age (weeks)	Diagnostic criteria for GDM	OSA diagnostic criteria	Adjusted OR (95% CI)	Prevalence (OSA with GDM)	Prevalence (OSA in the control group)
Izci Balserak et al. (2020) [17]	Case–control study	92	Case group, 33.99 ± 7.44; control group, 34.01 ± 7.00	AHI ≥ 5	24–36	GTT	PSG	4.71 (1.05, 21.04)	22%	9%
Facco et al. (2017) [19]	Prospective, cohort study	3,132	36.3 ± 8.6 (5 ≤ AHI < 15) 45.6 ± 11.3 (AHI ≥ 15)	AHI ≥ 5	6–15	GTT	6-channel monitor	Early, 3.47 (1.95, 6.19); late, 2.79 ( 1.63, 4.77)	——	——
Bublitz MH et al. (2018) [20]	Prospective, cohort study	25	36.1	AHI ≥ 5	24–32	FPG	MediByte device	——	17%	——
Bisson M. et al. (2014) [21]	Case–control study	52	Case group, 29.9 ± 4.8; control group, 30.1 ± 4.0	AHI ≥ 5	24–32	GTT	PSG	1.90 (0.52, 6.88)	31%	20%
Facco et al. (2014) [22]	Prospective, observational study	128	32.8 ± 8.7	AHI ≥ 5	6–37	Self-report	Watch-PAT100	——	30–50%	——
Newbold R. et al. (2021) [27]	Randomized controlled trial	65	32.7 ± 6.9	AHI ≥ 10	24–34	72-h glucose monitoring	PSG	——	——	——
Wanitcharoenkul E. et al. (2017) [28]	Randomized controlled trial	82	28.9	AHI ≥ 5	24–32	GTT or FPG	Watch-PAT200	——	< 24 weeks 62.7%; ≥ 24 weeks 37.2%	——
Ghesquière L. et al. (2020) [29]	Prospective, cohort study	67	42.4 ± 6.2	AHI ≥ 5	24–32	——	PSG	——	43.3%	——
Reutrakul et al. (2013) [30]	Case–control study	45	Case group, 29.7 ± 7.5; control group, 37.4 ± 9.2	AHI ≥ 5	12–40	GTT	PSG	6.60 (1.15, 37.69)	73%	27%
Louis J. et al. (2012) [31]	Prospective, cohort study	161	OSA group, 46.8 ± 12.2; NO-OSA group, 38.1 ± 7.5	AHI ≥ 5	< 37	GTT or FPG	PSG	——	15.4%	——
Facco FL. et al. (2014) [32]	Prospective, cohort study	188	32.8 ± 8.7	AHI ≥ 5	6–37	GTT	Watch-PAT100	mild OSA 1.5 (0.4, 6.0), moderate/severe OSA 3.6 (0.6, 21.8)	Mild, moderate, and severe OSA in early pregnancy: 21%, 6%, 3%	——

*OSA*, obstructive sleep apnea; *GDM*, gestational diabetes mellitus; *BMI*, body mass index; *OR*, odds ratio; *CI*, confidence interval; *CPAP*, continuous positive airway pressure; *AHI*, apnea–hypopnea index; *PSG*, polysomnography; *GTT*, glucose tolerance test; *FPG*, fasting plasma glucose

**Table 2:** The effect of OSA treatment on GDM

Reference	Type of study	Number of subjects	Mean ± SD of BMI	Diagnostic criteria for OSA (events/h)	Gestational age (weeks)	Diagnostic criteria for GDM	OSA diagnostic criteria	CPAP effect on glucose mean (95% CI)	CPAP treatment time
Chirakalwasan et al. (2018) [26]	Randomized controlled trial	36	30.4 ± 4.8	AHI ≥ 5	24–34	GTT	Watch-PAT200	− 0.29 (− 0.62, 0.04)	2 weeks, 3.39 ± 2.13 h/night

*OSA*, obstructive sleep apnea; *GDM*, gestational diabetes mellitus; *BMI*, body mass index; *CI*, confidence interval; *CPAP*, continuous positive airway pressure; *AHI*, apnea–hypopnea index; *GTT*, glucose tolerance test

### Prevalence of OSA with GDM

GDM occurred in 4.1% of women without pre-gestational diabetes [19]. Women with OSA developed GDM during pregnancy more often [29]. A study suggested that the prevalence of GDM in pregnant women with OSA is higher than that without OSA: 48.3% compared with 23.7% [29]. It is reported that the prevalence of OSA with GDM is 4.1–73% and is increasing year by year [19, 28, 30, 31]. In addition, the prevalence of OSA with GDM presents 30–50% in different stages of pregnancy [33]. Facco FL. et al. considered that the prevalence of GDM in severe OSA was 35% in the third trimester and 21% in early pregnancy [32]. However, Wanitcharoenkul E. suggested that the prevalence of GDM with OSA was 62.7% in early pregnancy and 37.2% in late pregnancy, respectively [28]. Moreover, the prevalence of GDM with OSA was 17% in a cohort consisting mainly of multigravida, multiparous, Caucasian women with GDM [20]. Accordingly, the prevalence of GDM with OSA varies widely in different studies.

### The Association Between OSA and GDM

Studies indicated the risk that the GDM accompanying with OSA was much higher [20, 21]. It is reported that GDM risk was significantly higher among women with a higher overall apnea–hypopnea index (AHI) [odds ratio (OR), 1.81; 95% CI, 1.01–3.27], higher AHI in REM (OR, 2.09; 95% CI, 1.02–4.31), and higher oxygen desaturation index (OR, 2.21; 95% CI, 1.03–4.73) [17]. Consequently, there is an association between GDM and OSA [17, 30]. For the presence and absence of OSA, the adjusted OR (aOR) for GDM was 3.47 (95% CI, 1.95–6.19) in early pregnancy [19]. In the second trimester of pregnancy, the severity of OSA was significantly associated with an increased risk of GDM even when the apnea–hypopnea index (AHI) was 1–5 events/h, which was below the standard threshold for OSA in non-pregnant women [21]. Additionally, the severity of OSA is correlated with IR, fasting glucose, and β-cell function [28]. Moreover, Chirakalwasan et al. explored the CPAP effect on GDM, and the results showed that β cell function was significantly improved and a trend of improving fasting blood glucose levels was found in GDM adherent to CPAP in an RCT [26]. These results support that the OSA plays a vital role in GDM.

OSA is a complex sleep disorder including IH and reoccurrence of arousal, leading to sleep fragmentation, light sleep, low amounts of SWS, and usually reducing total sleep time [34, 35]. A randomized cross-sectional study indicated that there was an approximate 30% reduction of cellular insulin sensitivity in adipocytes from subcutaneous fat samples collected in healthy subjects after 4 nights of sleep restriction compared with 4 nights of normal sleep, leading to impaired insulin signal transduction and IR in human fat cells [36]. Reutrakul et al. [30] evaluated OSA in women with GDM using PSG, and the results showed that the sleep fragmentation degree and AHI in women with GDM was higher than that in normal pregnant women. Authors suggested that sleep fragmentation and SWS inhibition led to a decrease in insulin sensitivity [37, 38]. In addition, acute exposure to IH in healthy volunteers was associated with decreased insulin sensitivity and impaired glucose tolerance [39]. Moreover, higher arousal index and more frequent hypoxic desaturation events are associated with higher fasting blood glucose levels [38]. On the contrary, two studies suggested that there is no relationship between GDM and OSA [21, 32]. However, the BMI effect on OSA or GDM has not been completely eliminated in these studies. Consequently, there is an association between GDM and OSA [19, 40].

Obesity may be a confounding factor of the association between OSA and GDM. However, Bourjeily et al. confirmed that OSA was associated with GDM (aOR, 1.51; 95% CI, 1.34–1.72) after adjusting for potential confounding factors (maternal obesity, pre-pregnancy hypertension, pre-pregnancy diabetes, maternal age, race/ethnicity, multiple births, tobacco use, alcohol use, drug use, rural/urban status, coronary heart disease, anemia, hyperlipidemia, hypothyroidism, disorders of the adrenal gland) in a study in which 1,577,632 deliveries women were included [40]. Moreover, GDM was found to be associated with the OSA (aOR, 6.60; 95% CI, 1.15–37.96) after adjusting for pre-pregnancy BMI though the sample size was small in this case–control study [30]. After adjusting for potential confounding factors including age, gestational age, BMI, and race, another study suggested that women with OSA had a higher GDM risk (OR, 4.71; 95% CI, 1.05–21.04) and GDM risk was also significantly higher among women with higher AHI (OR, 1.81; 95% CI, 1.01–3.27) [17]. Consequently, there is an association between GDM and OSA after adjusting for age, BMI, chronic hypertension, and pregnancy-related weight gain or not [19, 30, 40]. In conclusion, there is an association between OSA and GDM, which may not be caused by obesity.

### The Mechanisms of Association Between OSA and GDM

**Fig. 2:**
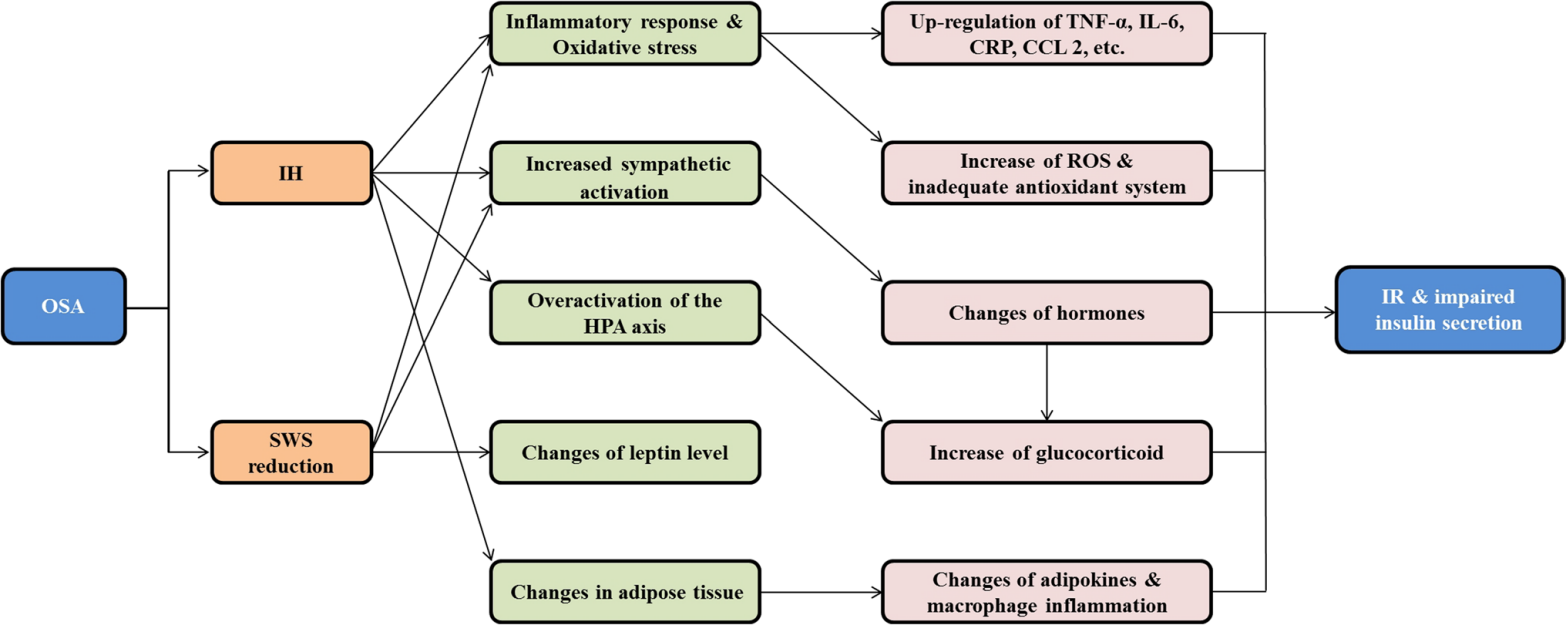
The mechanism of gestational diabetes mellitus and insulin resistance induced by obstructive sleep apnea. *OSA*, obstructive sleep apnea; *IH*, intermittent hypoxemia; *SWS*, slow-wave sleep; *HPA*, hypothalamic–pituitary–adrenal; *TNF-a*, tumor necrosis factor α; *IL-6*, interleukin-6; *CRP*, C-reactive protein; *CCL2*, CC chemokine family ligand 2; *ROS*, reactive oxygen species; *IR*, insulin resistance

Normal pregnant women usually have mild IR resulting from changes of hormonal or alteration of endothelial function during pregnancy [41]. Therefore, even small changes in sleep parameters may make pregnant women to be more susceptible to hyperglycemia or GDM. GDM has the same risk factors and genetic susceptibility as type 2 diabetes, which is related to IR and impaired insulin secretion [41]. Though exact mechanisms of the association between OSA and GDM remain not completely clear, several mechanisms may be involved in this association (Fig. 2).

#### Oxidative Stress and Inflammation

Increased studies suggest that oxidative stress and inflammation are associated with endothelial dysfunction [18, 42–44]. For pregnant women with OSA, IH has a range of downstream effects on tissues and organs [33, 45]. These effects include increased oxidative stress, the release of pro-inflammatory, and activation of cytokines or transcription factors [46–48]. IH during OSA leads to the increase of oxidative stress, which activates the pro-inflammatory cascade and the inflammatory pathway, consequently causing dyslipidemia and IR [47, 49]. In addition, cyclic reoxygenation after IH also promotes the production of reactive oxygen species (ROS) in OSA [50]. When ROS is accumulated, it will be eliminated by antioxidants [51]. However, excessive ROS that exceeds the antioxidant scavenging capacity often impairs the function of cells [52]. It is suggested that pancreatic cells are more vulnerable to oxygen stress than muscles, kidneys, and liver, which may be due to an inadequate antioxidant system [53]. Additionally, redox-sensitive transcription factors, such as nuclear factor kappa B (NF-κB) and HIF-1, are usually induced by ROS [51, 54]. The activation of NF-κB induces the release of several cytokines, such as tumor necrosis factor α (TNF-α) and interleukin-1 (IL-1), thereby leading to the presence of inflammation [51, 55]. Moreover, it is reported that IH upregulates CC chemokine family ligand 2 (CCL2) [56]. CCL2 is one of the key chemokines that regulate monocyte/macrophage migration and infiltration. Overexpressing CCL2 attracts inflammatory cells from the blood into adipose tissue and increases the number of macrophages, which cause metabolic phenotype to be deteriorated and then IR presents [57–59]. Consequently, the oxidative stress and inflammation induced by IH in OSA is an important factor in the pathogenesis of GDM.

Moreover, the reduction of SWS which presents in OSA may enhance the inflammation by increasing the concentration of TNF-α, interleukin-6 (IL-6), and C-reactive protein (CRP) in circulation, which is involved in IR [36, 60]. In the Cleveland Family Study, the reduction of sleep duration was also associated with increased TNF-α levels and increased habitual sleep time was associated with increased CRP and IL-6 levels [61]. Similarly, in the second and third trimesters, lower SWS and poorer sleep quality were found to be associated with higher levels of IL-6 [62]. TNF-α increases the phosphorylation of serine 307 of IRS-1 through JNK activation, which negatively modulates the interaction of stimuli with insulin receptors and the subsequent tyrosine phosphorylation of IRS-1, leading to impaired insulin signaling and β cell function [63–65]. OSA leads to pancreatic β-cell dysfunction, which is manifested by impaired basal insulin secretion and leads to diabetes [66]. TNF-α could significantly increase the secretion of IL-6, while IL-6 could reduce glucose transport [67]. In addition, IL-6 treatment increases insulin-stimulated glucose disposal, glucose uptake, and fatty acid oxidation in vitro via AMP-activated protein kinase [68]. Thus, the oxidative stress and inflammation induced by changes in sleep structure in OSA is a vital factor in the pathogenesis of GDM.

#### Increase of Sympathetic Activity

In the general population, frequent arousal and hypoxia generally reduce SWS time and increase sympathetic activity, leading to wakefulness during sleep [47, 69–71]. The reduction of SWS time increases the brain’s utilization of glucose and sympathetic nerve activity, which affect the regulation of glucose. In addition, when the sleep/wake cycle is abnormal, dozing may disturb the changes in hormones which regulate glucose metabolism, appetite, and the balance between sympathetic and parasympathetic nerves, which may impair glucose tolerance [72, 73]. Consequently, increased sympathetic activation may lead to GDM [47, 74].

#### Overactivation of the Hypothalamic–Pituitary–Adrenal (HPA) Axis

The HPA axis consists of three cell populations and specific hormones secreted by each group cell. Neurons in the medial paraventricular cells of the hypothalamic paraventricular nucleus (PVN) secrete corticotropin-releasing hormone (CRH), the endocrine cells of the anterior pituitary secrete adrenocorticotropic hormone (ACTH), and the endocrine cells in the adrenal cortical bundle mainly secrete the corticosteroid [75–77]. HPA activation stimulates the synthesis and release of ACTH, and the ACTH subsequently promotes gluconeogenesis and regulates blood glucose levels [78]. It is reported that poor sleep quality in pregnancy is associated with elevated levels of nocturnal cortisol [79], which suggested that the effect of OSA on the HPA axis may be related to sleep deprivation. In addition, pro-inflammatory cytokines and IH may lead to excessive activation of the HPA axis, which subsequently increase the release of glucocorticoid [80–82]. Long-term augment of the glucocorticoid increases susceptibility to impaired IR and impaired glucose tolerance [83]. In addition, there are significant negative correlations between morning plasma cortisol levels and AHI, as well as oxygen desaturation index, which confirm that OSA is associated with dysregulation of the HPA axis and alterations in glucose metabolism with increased risk for diabetes [84]. Moreover, sleep disruption and IH during sleep in pregnant women with OSA may lead to activation of the HPA axis and increase cortisol levels, consequently increasing the risk of GDM [85]. Therefore, the hyperactivation of the HPA axis caused by sleep fragmentation, pro-inflammatory cytokines, and IH may play a vital role in the development of GDM in patients with OSA.

CRH is a hypothalamic neuropeptide which is produced and released from the placenta at intervals and plays a central role in regulating the HPA axis [86, 87]. Studies confirmed the relationship between OSA and placental dysfunction caused by hypoxia damage in OSA [88–90]. Consequently, the association between OSA with GDM may be mediated by the placenta regulating the HPA axis.

#### The Levels of Leptin

Increasing evidence suggest that sleep deprivation, especially SWS loss, regulates appetite and satiety by reducing leptin sensitivity and increasing ghrelin levels, consequently boosting food intake and increasing IR [91, 92]. Leptin levels are higher in extreme situations where sleep time is shorter or longer [93]. Additionally, leptin levels were elevated in OSA and decreased after CPAP treatment, accompanying the increase of insulin secretion [94, 95]. Meanwhile, leptin is significantly associated with IR in patients with moderate-to-severe OSA [96]. However, the leptin levels in GDM are controversial. Some authors suggested that leptin levels were elevated in GDM and were associated with GDM status [97, 98], while other authors found there were no changes or reductions in leptin levels of GDM [99, 100]. Consequently, more studies are needed to confirm the role of leptin in the association between OSA and GDM.

#### Adipose Tissue

Obesity is associated with the presence of OSA and diabetes [101]. Obesity and particularly central adiposity are potent risk factors for sleep apnea [102]. At the same time, obesity in pregnancy is associated with the occurrence of GDM [103]. It seems that the increased prevalence of OSA in GDM patients results from obesity rather than the association between OSA and GDM. However, several studies confirm that OSA is still associated with GDM after adjusting for BMI [17, 30, 40], which means that obesity is not really the main factor contributing to the association between GDM and OSA. Authors suggested the maternal TNF-α level in circulation increased and was an independent predictor of GDM [104, 105]. Additionally, changes of adipokines induced by IH aggravate inflammation in adipose tissue, thereby leading to IR [106, 107]. Meanwhile, overexpression of monocyte-chemoattractant protein-1 (MCP-1) promotes the infiltration of monocytes/macrophages into adipose tissue and activates pro-inflammatory macrophages which are related to IR [108, 109]. Moreover, the increased free fatty acids (FFA) induce macrophages to produce inflammatory cytokines by activating the NF-κB pathway, which is related to IR [110, 111]. More importantly, pregnant women with OSA present an augment of the NF-κB pathway as well as macrophages inflammation [47, 112]. Thus, inflammation of macrophages in adipose tissue of the OSA may be related to GDM. However, macrophages inflammation and its signal pathways in OSA with GDM remain unclear and require more research to confirm in the future.

### The Effect of OSA Treatment on GDM

CPAP is the gold standard for the treatment of OSA and can reduce the occurrence of various complications [113, 114]. However, the effect of CPAP on glucose metabolism and adverse outcomes of pregnancy is still debated. The authors suggested that CPAP treatment of OSA significantly improved glycaemic control via amelioration of evening fasting glucose metabolism and a reduction in the dawn phenomenon and may be more beneficial in participants with poor glycemic control at baseline [115, 116]. In addition, CPAP is beneficial to the decrease of glycated hemoglobin and improves β cell function [117]. On the contrary, other authors suggest that therapeutic CPAP does not significantly improve glycaemic control or IR [118, 119].

Carnelio S. confirmed that CPAP does not prevent adverse outcomes of pregnant women (early miscarriage, premature deliveries, etc.) [120]. However, among participants who adhered to CPAP, there is a significant improvement in the hospitalization rates of premature births and unplanned cesarean sections and neonatal intensive care units were lower in mothers who used CPAP for > 2 weeks than those who used CPAP for ≤ 2 weeks [26, 121].

CPAP is a safe and effective method for pregnant women with OSA [122, 123]. It was reported that 2 weeks of CPAP treatment in late pregnancy was safe but did not improve glucose metabolism of OSA with GDM, though there was a tendency of improvement in fasting glucose [26]. However, CPAP treatment improved insulin secretion (*P* = 0.002) and insulin sensitivity of OSA with GDM (*P* = 0.015) after dealing with nonadherence in the RCT [26]. At present, it is uncertain whether the CPAP is beneficial to OSA with GDM because a single study with a small sample size (*n* = 36) was included in this review [26]. Accordingly, more research on CPAP effects on OSA with GDM are urgently needed.

## Discussions

Currently, most of the studies suggest that there is an association between OSA and GDM. However, the exact mechanisms of this association remain unclear. There are several possible mechanisms involved in this association. Among these mechanisms, the IH and reduction of SWS were plausible. On the one hand, IH in OSA induces the products of oxidative stress and inflammation in adipose tissue or circulation, which lead to IR or diabetes [47, 49]. On the other hand, IH results in dysregulation of the HPA axis and thereby impairs glucose metabolism [80, 81]. In addition, SWS reduction plays an important role in the association between OSA and GDM. SWS reduction in OSA enhances the inflammation by increasing the inflammatory cytokines, increases the sympathetic activation, and causes the changes in leptin level, which result in the development of GDM [36, 60, 72, 73, 91].

Obesity hypoventilation syndrome (OHS) may develop from a complex interaction between severe OSA, central obesity, obesity-related respiratory disorders, and reduced respiratory drive [124]. OSA is present in 90% of individuals with OHS, and the remaining 10% of OHS is characterized by an apnea–hypopnea index (AHI) < 5 events/hour [125]. Currently, OHS is still poorly understood [126]. Most previous studies on OSA did not distinguish OHS from OSA, which means that the OSA subjects in these studies may have mixed with OHS patients. Similarly, we failed to separate OHS from OSA in this review due to the limitations of previous studies. Therefore, studies on the association between OSA and GDM are needed with the exclusion of OHS confounding in the future.

Moreover, studies are needed to explore the mechanisms of association between OSA and GDM. IH may up-regulate other factors besides resistin, TNF-α, and CCL2. Cytokines or inflammatory factors which strongly contribute to GDM are needed to find. Additionally, the insulin downstream signal of insulin regulated by cytokines remains unclear. Meanwhile, the effect of leptin levels in GDM is still debated. In addition, clinical trials should be designed to confirm the effect of CPAP on the maternal and fetal outcomes of GDM.

There were several limitations in this review. Firstly, some studies included presented a small sample size. Secondly, studies included failed to distinguish OHS from OSA. Thirdly, among the twelve studies included, there are only three RCTs. Another limitation is that none of the included studies listed the type of obesity and gestational weight gain, which may be a confounding factor. However, these limitations pointed to the direction of future research. Meanwhile, several strengths were presented in this review. In addition, the studies in which OSA subjects diagnosed with a questionnaire were excluded.

In conclusion, researchers should pay more attention to the association between OSA and GDM in the future. More studies should focus on mechanisms of this association as well as the CPAP effects on the OSA with GDM.

## Data Availability

Not applicable.
